# Therapeutic challenges of a renal collision tumour comprising clear cell renal cell carcinoma and metastatic breast carcinoma: a case report

**DOI:** 10.1038/s44276-026-00233-x

**Published:** 2026-08-03

**Authors:** AJP Fulton, AY Warren, S. Appukutty, A. Matakidou, W. Ince, B. O’Carrigan, K. Fife, T. Eisen, A. Shakur, GD Stewart, K. McAdam, JO Jones

**Affiliations:** 1 Cambridge University NHS Foundation Trust, Cambridge, UK; 2 Department of Oncology, University of Cambridge, Cambridge, UK; 3 Department of Pathology, University of Cambridge, Cambridge, UK; 4 Department of Surgery, University of Cambridge, Cambridge, UK

## Abstract

Collision tumours, defined by the coexistence of two histologically distinct neoplasms within a single organ, are rare and typically arise from different cell lineages. We report a unique case of a 64-year-old woman with a history of oestrogen receptor (ER)–positive, HER2-negative breast carcinoma who developed a renal mass later identified as a collision tumour comprising clear cell renal cell carcinoma (ccRCC) and metastatic breast carcinoma. Initial imaging and biopsy suggested ccRCC alone; however, post-nephrectomy histopathology revealed metastatic breast carcinoma foci within the ccRCC and renal sinus fat, confirmed by ER, GATA3, and mammaglobin positivity. This case highlights diagnostic challenges in distinguishing synchronous primaries from metastases, particularly where biopsy sampling may obscure dual pathology. Management required balancing treatment of localised ccRCC with systemic therapy for metastatic breast cancer. Multidisciplinary evaluation is essential to guide optimal care.

## Introduction

Collision tumours are rare entities defined by the coexistence of two histologically distinct neoplasms in the same organ. Within the kidney, collision tumours most often involve combinations of different subtypes of renal cell carcinoma [1]. The coexistence of a primary renal neoplasm with a metastasis from another primary site is exceptionally uncommon.

Breast carcinoma metastasising to the kidney is uncommon, and its coexistence with a primary clear cell renal cell carcinoma (ccRCC) in the same renal lesion is exceptionally rare. Such presentations pose diagnostic and therapeutic challenges, particularly in distinguishing the metastasis from a synchronous primary tumour, as management strategies differ markedly between these two scenarios.

We report a case of a patient with metastatic hormone receptor–positive breast carcinoma who developed a renal mass subsequently found to represent a collision tumour composed of ccRCC and metastatic breast carcinoma.

## Results

### Clinical background

A 64-year-old female with a background of sickle cell trait and a prior oestrogen receptor (ER) positive, human epidermal growth factor receptor 2 (HER2) negative right breast carcinoma was reviewed in the renal oncology clinic for a new renal mass. At the time of her initial breast cancer diagnosis in 2002, she received neoadjuvant doxorubicin and paclitaxel chemotherapy, followed by a right mastectomy and axillary node clearance. Adjuvant radiotherapy was completed in June 2002, and she remained disease-free for over two decades.

### Clinical course

**Fig. 1:**
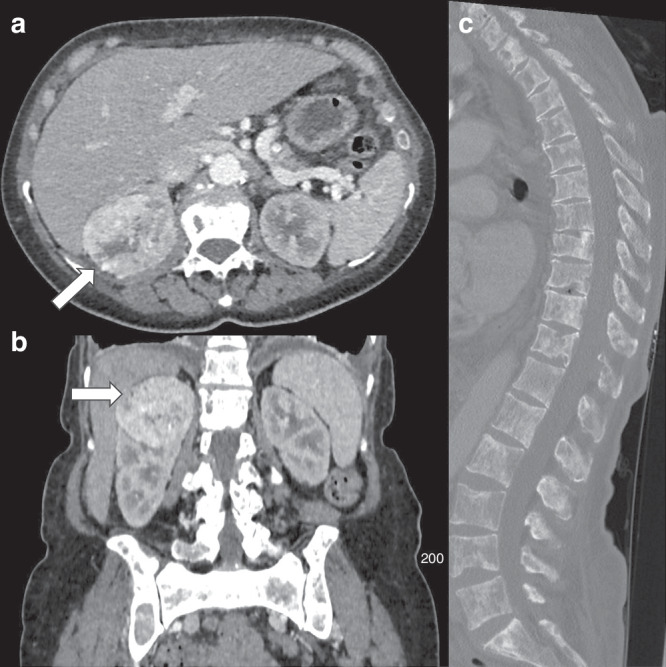
CT imaging demonstrating a right upper pole renal mass with diff use mixed sclerotic and lytic bone lesions,radiologically favouring metastatic breast cancer. Axial (**a**) and coronal (**b**) images from post contrast CT of the abdomen demonstrates a heterogeneously enhancing right upper pole renal mass (white arrows). Sagittal reconstruction of the spine (**c**) demonstrates diffuse mixed sclerotic and lytic bone lesions, more typical of breast cancer metastases.

In early 2025, she presented with progressive generalized pain initially attributed to fibromyalgia. Routine blood tests demonstrated a normocytic anaemia and an elevated alkaline phosphatase level. Further evaluation with cross-sectional imaging revealed multiple mixed lytic and sclerotic bone lesions and an incidental 65 mm right renal mass (cT1b as per UICC TNM 8th edition) (Fig. 1). She was initially investigated on a myeloma pathway, but serum electrophoresis, immunofixation, and free light chain assays were unremarkable. The renal mass was subsequently evaluated by the urology team and a percutaneous biopsy confirmed grade 2 clear cell renal cell carcinoma (ccRCC).

Bone metastases from RCC are typically lytic, whereas mixed lytic and sclerotic bone lesions are far more common in metastatic breast cancer. The case was therefore discussed at the regional multidisciplinary team (MDT) meeting given the above. It was considered that the bone lesions would most likely represent recurrence of her prior breast malignancy. Subsequent CA15-3 testing demonstrated an elevated result of 1215 kU/L, in keeping with likely breast carcinoma metastases. A targeted bone biopsy was therefore performed, which demonstrated metastatic adenocarcinoma consistent with recurrent breast carcinoma, ER positive and HER2 negative.

A diagnosis of metastatic breast carcinoma and a synchronous right kidney renal cell carcinoma was therefore made. The patient commenced first-line systemic therapy with letrozole and ribociclib. Given the confirmed clear cell RCC was cT1b, she was referred for surgical resection.

### Histopathology findings

On 21st August 2025, she underwent a robot assisted laparoscopic radical nephrectomy. Histopathological examination revealed a 60 mm grade 3 clear cell renal cell carcinoma without sarcomatoid or rhabdoid morphology. Formal staging was reported as pT3a pNx(UICC TNM 8th Edition) with a Leibovich score of 6/11. Resection margins, including the renal vein margin, were clear.

**Fig. 2:**
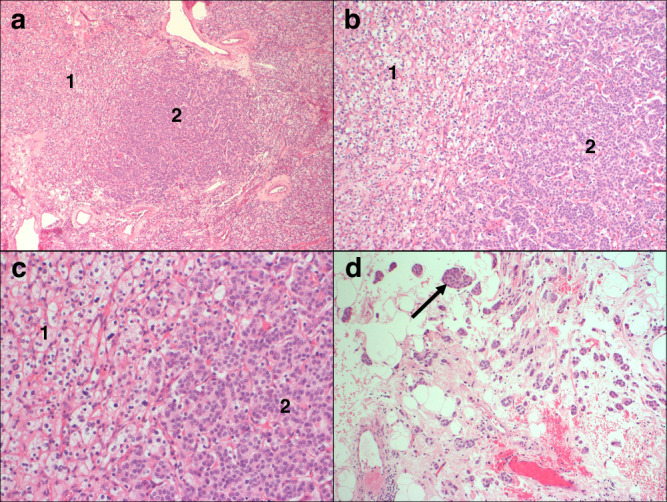
Hematoxylin and eosin (H&E)–stained sections of the collision tumour at varying magnifications. Images (**a**–**c**) show clear cell renal cell carcinoma (ccRCC) (1) surrounding metastatic breast carcinoma (2) at ×20 (**a**), ×100 (**b**), and ×200 (**c**) magnification, respectively. Image (**d**) demonstrates metastatic breast carcinoma deposits within the renal sinus fat at 100× magnification.

**Fig. 3:**
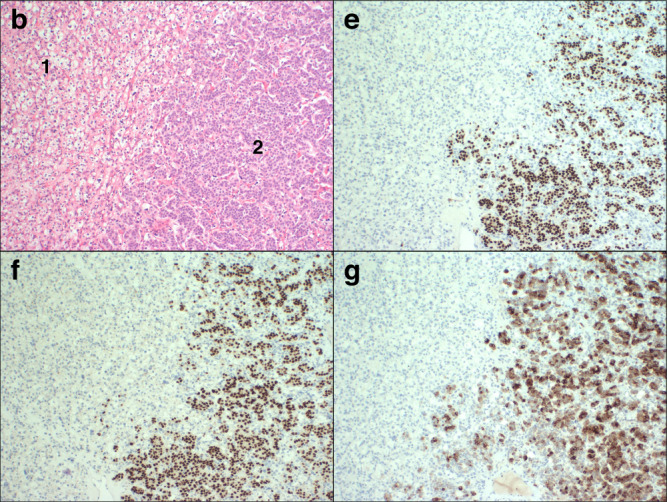
Hematoxylin and eosin (H&E) and immunohistochemistry (IHC)–stained sections of the collision tumour at ×100 magnification. Image (**b**) shows the H&E-stained section of the collision tumour. IHC staining confirms breast carcinoma origin, with tumour cells demonstrating positivity for oestrogen receptor (ER) (**e**), GATA3 (**f**), and mammaglobin (**g**).

Within the ccRCC tumour and the renal sinus fat, there were multiple small incidental foci of metastatic breast adenocarcinoma. Morphologically, these foci were composed of nests of cells and tubules/glands. The tumour cells had round/oval vesicular nuclei with inconspicuous nucleoli and a moderate amount of pale eosinophilic cytoplasm. Immunohistochemistry supported a breast origin, with tumour cells demonstrating positivity for ER, GATA3, mammaglobin and AE1/3. Markers of renal or alternative primary origins were negative, including Pax-8, WT1, TTF1, CK7, CK5/6, CDX2, and neuroendocrine markers (chromogranin-A and synaptophysin). This immunophenotype therefore confirmed metastatic breast carcinoma (Figs. 2 and 3).

### Management plan

The patient had been commenced on treatment for metastatic ER positive, HER2 negative breast cancer with a combination of letrozole and ribociclib prior to her renal surgery. Ribociclib was paused over the perioperative period and was recommenced once she had recovered post nephrectomy. Postoperatively, she was reviewed in the renal oncology clinic to discuss the potential role of adjuvant pembrolizumab for high-risk localised renal cancer treatment. This adjuvant renal cancer treatment was under consideration given her likely durable response to first line ER positive breast cancer treatment [2]. The anticipated benefits and possible toxicities of Pembrolizumab were carefully reviewed, alongside the need to continue systemic therapy for breast cancer. Following a shared decision-making discussion, the patient elected for active surveillance for any recurrence of her renal cell carcinoma and continuation of breast cancer management with ribociclib and letrozole. Denosumab was also initiated in view of bone metastases. She is currently tolerating treatment well, and follow-up CT imaging is awaited to assess response.

## Discussion

Collision tumours represent the co-existence of two histologically distinct neoplasms within a single organ, without histologic mixture or transition. While they have been reported in various organs, their occurrence in the kidney most often involves dual primary renal cell carcinomas or combinations of renal and urothelial neoplasms [1]. In contrast, tumour-to-tumour metastasis describes the phenomenon whereby one neoplasm (the recipient tumour) harbours metastatic deposits from another primary malignancy within its substance. Isolated renal metastases, meanwhile, occur in the absence of a synchronous primary renal tumour. While there is some overlap in terminology within the literature, the present case demonstrates features most consistent with a collision tumour, given the presence of spatially distinct tumour components within the same lesion.

The coexistence of a primary renal cell carcinoma and metastatic breast carcinoma within the same renal mass is exceedingly rare, with only isolated case reports describing breast metastases in renal tumours [3]. In contrast, two coexisting primary tumours is much more common [4]. To our knowledge, this is the first reported case of a renal collision tumour comprising clear cell renal cell carcinoma (ccRCC) and metastatic oestrogen receptor (ER)–positive, HER2–negative breast carcinoma late recurrence.

The pathogenesis of collision tumours remains incompletely understood. For synchronous tumours arising from a common histological background, several mechanisms have been proposed. This includes microenvironment alteration by one tumour facilitating the development of the other, differentiation from a common precursor or independent evolution in adjacent cells due to shared common oncogenic stimuli [5, 6]. For collision tumours that consist of different primary tissues, microenvironment features are felt to provide a selective advantage for metastasis development [7].

In the present case, metastatic breast carcinoma foci were embedded within the ccRCC and renal sinus fat. A number of lipid metabolites are known to be selective oestrogen receptor modulators that both in vivo and in vitro have demonstrated the ability to enhance the proliferation of ER positive breast carcinomas [8]. As such, the highly vascular nature and lipid-rich microenvironment of ccRCC may provide a fertile niche for metastatic seeding.

From a diagnostic perspective, this case highlights the importance of maintaining a broad differential diagnosis when evaluating renal masses in patients with prior malignancies. Radiologically, distinguishing a metastatic lesion from a primary renal neoplasm can be challenging. Percutaneous biopsy provides valuable histological confirmation but may be limited by sampling error, as metastatic foci may represent only a minor component within the dominant tumour mass. Radiological heterogeneity within a renal mass may occasionally suggest mixed pathology and could prompt targeted or multi-region biopsy. Similarly, multi-core biopsy strategies may theoretically improve detection of minor tumour components. However, in practice, metastatic foci may represent only a small proportion of the lesion, limiting the sensitivity of these approaches whilst increasing risks related to the procedure. Multidisciplinary discussion incorporating histopathology, radiology, and clinical context is therefore essential.

Therapeutically, the presence of two distinct malignancies within one organ complicates management decisions. ccRCC is primarily treated surgically when localised, whereas metastatic breast carcinoma is managed systemically. In this case, nephrectomy was undertaken both for local control and to obtain definitive histopathological characterisation. Post-operatively, systemic therapy with a CDK4/6 inhibitor and aromatase inhibitor was continued for metastatic ER positive breast cancer. The potential role of adjuvant pembrolizumab for the ccRCC component was considered, as a Leibovich score of 6/11 placed the patient at intermediate–high risk of recurrence. However, concerns have been raised about immunotherapy toxicity & overtreatment in this setting [9]. In addition, combining immune checkpoint inhibitors with CDK4/6 inhibitors raises concerns regarding overlapping toxicities, particularly hepatotoxicity and pneumonitis. Importantly, the presence of active metastatic breast cancer significantly influenced the overall risk–benefit assessment. Following multidisciplinary discussion and shared decision-making, active surveillance was therefore favoured. Early-phase data in ER positive breast cancer combining CDK4/6 inhibition with pembrolizumab suggest mixed safety profiles and uncertain synergistic efficacy [10, 11]. This underscores the need for caution and multidisciplinary evaluation and informed decision making before concurrent administration in such collision tumours.

## Conclusion

This case underscores the necessity of individualised treatment planning when diagnosing and managing synchronous malignancies with divergent biological behaviours and therapeutic paradigms. It also illustrates the evolving landscape of cancer survivorship, wherein patients successfully treated for an initial malignancy may present years later with new or metastatic disease, occasionally in anatomically and biologically unexpected combinations. Further molecular characterisation of such collision tumours, including genomic or transcriptomic profiling, may provide insight into shared pathways of tumour progression, microenvironmental permissiveness, and potential therapeutic vulnerabilities. This may help to inform optimal management strategies in these rare cases and help to minimise potential toxicities from combination approaches.

## Methods

This case was identified during multidisciplinary review at Cambridge University Hospitals NHS Foundation Trust, Cambridge, UK. Clinical data, imaging, histopathological findings, and treatment details were obtained from electronic medical records and institutional databases. All information was anonymised prior to analysis and reporting.

Formalin-fixed paraffin-embedded (FFPE) tissue from the nephrectomy specimen was examined using haematoxylin and eosin staining and a panel of immunohistochemical markers, including ER, GATA3, mammaglobin, CK7, Pax-8, and WT1, performed according to standard diagnostic laboratory protocols. Histopathological review was undertaken by consultant pathologists to confirm the coexistence of distinct tumour components. Cross-sectional imaging was reviewed by specialist radiologists, and representative images were included to illustrate key findings.

The case was discussed at a regional oncology–urology multidisciplinary team (MDT) meeting to guide diagnosis and management.

## Data Availability

All data supporting the findings of this report are included within the article. Additional anonymised clinical or histopathological information can be made available from the corresponding author upon reasonable request, in accordance with institutional and ethical guidelines.
